# Retraction Note: Evaluating the role of propolis and bee venom on the oxidative stress induced by gamma rays in rats

**DOI:** 10.1038/s41598-024-59116-1

**Published:** 2024-04-17

**Authors:** Eithar K. El Adham, Amal I. Hassan, M. M. A. Dawoud

**Affiliations:** Radioisotopes Department, Nuclear Research Center, Egyptian Atomic Energy Authority, Dokki, Giza, 12311 Egypt

Retraction of: *Scientific Reports* 10.1038/s41598-022-05979-1, published online 16 February 2022

The Editors retracted this Article.

After publication, concerns have been raised about some of the data shown in the Article, in particular that some of the images overlap and show signs of inappropriate image manipulation. Specifically:

• Panel d of Figure 6 appears to overlap with panel h of the same figure;

• A section of the left half of Panel g of Figure 8 appears to have been re-scaled and re-used in Panel h of the same figure;

• The top half of Panel k of Figure 9 appears to overlap with the bottom half of Panel o of the same figure.

Authors provided some of the original data, but these were insufficient to address the concerns. The Editors therefore no longer have confidence in the integrity of the data in this Article.

Amal I. Hassan disagrees to this retraction. Eithar K. El Adham and M. M. A. Dawoud have not replied to correspondence from the Editors.

